# 20 Autologous Meshed Split Thickness Graft Healing in Interstice versus Grafted Sites: A Histological Characterization

**DOI:** 10.1093/jbcr/irac012.024

**Published:** 2022-03-23

**Authors:** Mary A Oliver, Bonnie C Carney, John W Keyloun, Lesle M Jimenez, Lauren T Moffatt, Jeffrey W Shupp

**Affiliations:** Burn Center at MedStar Washington Hospital Center, Washington DC, District of Columbia; Medstar Health Research Institute, Washington DC, District of Columbia; Burn Center at MedStar Washington Hospital Center, Washington DC, District of Columbia; MedStar Health Research Institute, Washington, District of Columbia; Burn Center at MedStar Washington Hospital Center, Washington DC, District of Columbia; MedStar Washington Hospital Center, Washington DC, District of Columbia

## Abstract

**Introduction:**

Autologous skin grafting is a common technique used in the treatment of full thickness (FT) wounds to aid in healing, wound closure, and reducing the likelihood of developing hypertrophic scarring. Meshed split thickness skin grafts (mSTSG) which contain portions of the dermis and epidermis are the gold standard for the treatment of FT wounds because they allow for expansion of skin taken from a relatively small donor site. It has largely been hypothesized that in mSTSG skin progenitor cells migrate from the edges of healthy donor tissue to aid in healing the interstices created by meshing. For this reason, meshed wound healing is not homogenous as interstice and meshed sites display distinct healing characteristics. This study aims to characterize the differences between interstice and grafted site healing.

**Methods:**

Wound healing was evaluated *in vivo* using Duroc pigs. In this model, 4 animals had 10.16 cm by 10.16 cm full-thickness burns created on bilateral flanks for a total of 12 wounds. On day 2, the burns were excised down to subcutaneous tissue, mSTSG was harvested, meshed 4:1, and applied to the prepared wound beds. Wounds were photographed and sampled on days 5, 9 and, 15. Punch biopsies from either the grafted area or interstice area were taken at each time point, processed, and imaged. Images were used to quantify epidermal and dermal thickness, cellularity, and rete ridges.

**Results:**

Epidermal thickness at day 5 in interstices was significantly thinner than in graft (1.73±4.33µm vs. 75.7±66.1µm, p< 0.05). By day 9, interstice epidermal thickness was comparable to graft thickness (194.9±157.7µm vs. 199.5±117.7µm). On the other hand, dermal thickness was elevated in the interstice at days 9 (1850.4±642.0µm vs. 1277.6±652.0µm, p< 0.05) and 15 (2469.9±626.14µm vs. 1660.7±674.6µm, p< 0.01) to a significant degree. Cellularity was greater at all time points in the interstice compared to the grafted sites. Similarly, rete ridge ratios (RRR) were significantly greater in the grafted areas at day 5 (0.0±0.0µm v.s. 1.0±0.7µm, p< 0.01) and day 9 (1.32±1.2µm vs. 1.9±0.45µm, p< 0.05).

**Conclusions:**

These data show that within a grafted burn wound, healing is a dynamic and heterogenous process when looking at interstice and graft sites, respectively. Grafted sites were thinner throughout, showed decreased inflammatory cell infiltrate, and exhibited higher RRR. Thicker tissue layers and upregulated cellularity in interstices point to a wound healing trajectory that is slower than grafted sites, even by the time wounds are fully re-epithelized.

